# Idiopathic Benign Hyper-CK-Emia

**Published:** 2009-03

**Authors:** Prashant Kaushik, Anuradha Gonuguntla

**Affiliations:** 1 *Specialty Clinics, St. Alexius Medical Center, Bismarck, North Dakota, USA;*; 2 *Center for Family Medicine, Bismarck, North Dakota, USA*

**Keywords:** idiopathic, benign, hyper-CK-emia

## Abstract

It is not uncommon for a rheumatologist to get a request for evaluating a patient with persistent elevation of serum creatine (phospho) kinase (CK) level (hyper-CK-emia) especially if this laboratory abnormality is persistent. We present a descriptive analysis of 16 consecutive adult patients seen with a condition called idiopathic benign hyper-CK-emia at a tertiary care Rheumatology Clinic in Bismarck, North Dakota. This condition is well described in the medical literature but unfortunately is not recognized very often by the primary care physicians especially its benign course. A short review of literature is also presented. To the best of our knowledge this is the first such report on this condition from the state of North Dakota.

## INTRODUCTION

It is not uncommon for a rheumatologist to get a request for evaluating a patient with persistent elevation of serum creatine (phospho) kinase (CK) level especially if the degree of elevation is moderate to severe. The concerns in such a situation include inflammatory myopathy (polymyositis, dermatomyositis), metabolic myopathy (disorders of glycogen and lipid metabolism), inclusion body myositis and several neuropathies especially the hereditary varieties (Charcot-Marie-Tooth syndromes, facioscapulohumeral dystrophy, Duchenne muscular dystrophy) etc.

## STRUCTURE

Over a period of two years, we evaluated 16 Caucasian patients consecutively with persistent, modest (less than three times the upper limit of normal) elevation of CK level at a tertiary care referral center in Bismarck, North Dakota. All patients were evaluated (initially and on follow-up visits) by the same Rheumatologist (PK). Serum CK level was estimated using Beckman Coulter Synchron LX20 (enzymatic rate method) at the same laboratory for all patients. The data was analyzed using standard statistical methods.

## RESULTS

The age range of the patients was 27 to 47 years. Nine patients were male and seven were female. All patients were asymptomatic. All patients denied a history of neuropathy, myopathy or elevated serum CK levels among their first degree relatives. No evidence of neuropathy was noted on physical examination. None of the patients had evidence of proximal or distal muscle weakness in the extremities or the trunk on an MRC (Medical Research Council) scale (1). Other causes of an elevated CK (hypothyroidism, use of drugs like steroid/statin/fibrates/cyclosporine, rhabdomyolysis and polymyositis) were ruled out by appropriate history, physical examination and laboratory testing.

A forearm ischemic exercise test was normal and nerve conduction study combined with an electromyogram failed to reveal any type of underlying neuropathy or myopathy (inflammatory or non-inflammatory). Hence, a diagnosis of ‘idiopathic hyper-CK-emia’ (IH) was made.

## DISCUSSION

Persistent elevation of serum creatine kinase (CK) in individuals with normal neurological and laboratory examinations is called IH. It can be a familial condition in up to 46% of the cases. Familial IH is a benign genetically heterogeneous condition that is autosomal-dominant in at least 60% of cases, with a higher penetrance in men (2).

Patients with IH have a benign prognosis. A relatively recent study compared 11 patients with IH to 11 age-matched healthy controls. Results indicated that maximal and sub maximal bouts of dynamic exercise on a cycle ergometer did not lead to larger increases in serum CK activity or more complaints in patients with IH than in age-matched healthy controls. This suggested that exercise does not result in much more extensive muscle damage in patients with IH than in healthy subjects (3).

Another study has shown that caveolin-3 gene mutation could be the cause of IH. Even though immunohistochemical analysis in two affected patients strongly suggested that caveolin-3 was properly localized in the muscle tissue, it was suspected to be not functioning normally and could thus explain their persistent hyper-CK-emia (4).

## CONCLUSION

Long-term follow-up of patients with IH does not reveal clinical deterioration. So it seems justifiable to refrain from routine long-term follow-up in these patients (5). The results of this small longitudinal study need to be confirmed with a larger study.
